# Study of influence of *Catha edulis* (Khat) chewing on oral pharmacokinetics of irbesartan in rats using a newly developed HPLC-UV method

**DOI:** 10.1016/j.jsps.2022.01.002

**Published:** 2022-01-13

**Authors:** Hassan A. Alhazmi, Mustafa A. Bakri, Yahya A. Mohzari, Yousef G. Alshigaify, Mohammed Al Bratty, Sadique A. Javed, Asim Najmi, Ziaur Rehman, Waquar Ahsan, Manal Mohamed Elhassan Taha

**Affiliations:** a Department of Pharmaceutical Chemistry, College of Pharmacy, Jazan University, Jazan P.O. Box 114, 45142, Saudi Arabia; b Substance Abuse and Toxicity Research Centre, Jazan University, P.O. Box 114, 45142 Jazan, Saudi Arabia

**Keywords:** Khat, Irbesartan, HPLC, Pharmacokinetics, Cytochrome P450

## Abstract

Khat consumers might use a number of drugs for underlying conditions; however the potential drug-herb interaction between khat and other drugs including Irbesartan (IRB) is unknown. The present study was conducted to evaluate the effects of khat chewing on pharmacokinetic profile of IRB, a commonly available antihypertensive agent. The pharmacokinetic profile of orally administered IRB (15.5 mg/kg) with and without pre-administration of khat (12.4 mg/kg) were determined in Sprague-Dawley rats. IRB was estimated in rat plasma samples using a newly developed HPLC method. The chromatographic separation of the drug and internal standard (IS) was performed on a C-18 column (Raptor C-18, 100 mm × 4.6 mm id.; 5 µm) using a mobile phase consisting of 10 mM ammonium acetate buffer (pH 4.0) and acetonitrile in a ratio 60:40 *v/v*. Acceptable linearity for IRB was recorded at 1 – 12 µg/mL concentration range (R^2^ > 0.99). Intra-day and inter-day precision (%RSD = 0.44% − 3.27% and 0.39–1.98% respectively) and accuracy (% recovery = 98.3 – 104.3%) in rat plasma was within the acceptable limit according to USFDA guidelines. The AUC_0-t_ was found to be significantly increased in IRB-khat co-administered rats as compared to rats receiving IRB only; whereas, the T_max_ (0.5 h) value remained unchanged. Results of this study revealed that the IRB level considerably increased in rat plasma upon co-administration of khat. This might be due to the inhibition of CYP2D9 by khat which is the principal cytochrome P450 isoform responsible for IRB metabolism.

## 1 Introduction

Khat (*Catha edulis* [Vahl] endl), an evergreen shrub is native to Middle East and East Africa. The fresh leaves of the plant are chewed globally by millions of people, mainly belonging to these regions due to its mild amphetamine-like psycho-stimulant effect (Lim et al., 2019, Nichols et al., 2015). Cathine and cathinone alkaloids are the major active constituents of khat leaves that are chiefly responsible for its euphoric and stimulating effects (Dhaifalah and Šantavý, 2004). Approximately 10 million people across the globe consume khat leaves on daily basis and an average amount of 100–400 g fresh leaves are being chewed per person per day (Lim et al., 2019). Organ systems of the body, including cardiovascular, central nervous, reproductive and respiratory systems are potentially affected by long-term intake of khat leaves; however, both harmful as well as beneficial effects have been reported (Engidawork, 2017).

As a consequence of growing trend in the use of herbal medicines, investigation of drug-herb interaction is becoming extremely important, in case of concomitant administration of drugs and herbal products. Drug-herb interactions mainly occur when the phytoconstituents are capable to alter drug metabolism through modification of the expression or activity of metabolizing enzymes, influencing the pharmacokinetic profile and clinical efficacy of the drug. This may also result in serious adverse drug reactions associated with life-threatening events (Lim et al., 2019). A number of endogenous and exogenous compounds are metabolized by cytochrome P450 (CYP), a superfamily of enzymes, of which families 1, 2 and 3 are mainly responsible for biotransformation of xenobiotics including drugs, herbs and dietary chemicals. Approximately, 70–80% of the clinically used therapeutic agents are metabolized by CYP450 family enzymes. Among the CYP isoforms, CYP2D6 and CYP3A4 regulate the metabolism of most of the therapeutic agents, whereas, CYP2D9 and CYP3A4 are among the highest expressed isoforms. Other enzymes of the CYP family are involved in metabolizing biomolecules such as fatty acids and steroids (Lynch and Price, 2007, Amacher, 2010, Zanger and Schwab, 2013).

**Fig. 1:**
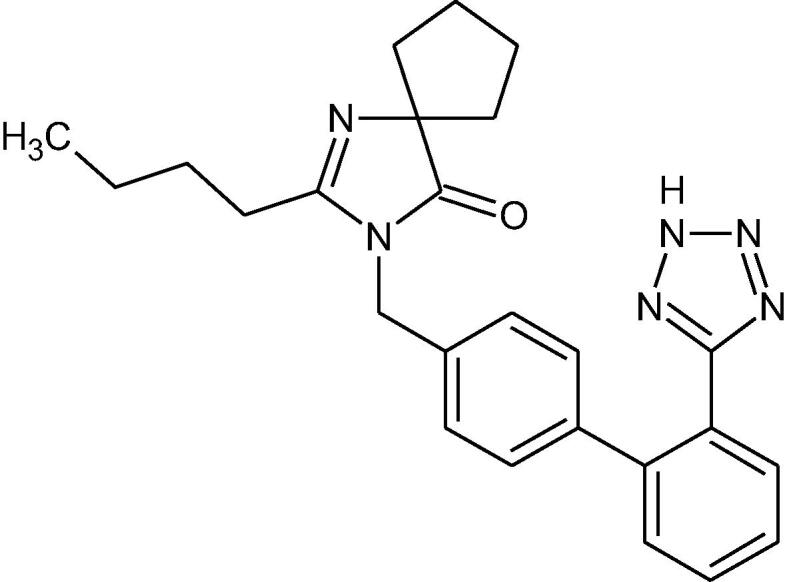
Chemical structure of IRB.

IRB, a biphenylyltetrazole derivative (Fig. 1), is a potent antihypertensive agent possessing a non-peptide chemical structure with strong lipophilic character and is practically insoluble in water (Cazaubon et al., 1993). It is a non-competitive, long-acting angiotensin II receptor antagonist, hindering the vasoconstrictive activity of angiotensin II through selective interaction with AT1 receptor subtype and resulting in the reduction of blood pressure (Ellis and Patterson, 1996, Powell et al., 1998, Borghi et al., 2006). IRB is absorbed well following oral administration and displays favorable pharmacokinetic and pharmacodynamic profiles required for a perfect antihypertensive agent. Following oral administration, IRB is absorbed rapidly (T_max_ = 1.5–2 h), showing impressive bioavailability of 60–80% (remained unaffected by foods), which is higher than other AT1 antagonists. It also has a prolonged elimination half-life (t_1/2_ = 11–15 h) providing a 24 h blood pressure control with a single daily oral dose of 150 or 300 mg (Marino et al., 1998, Ruilope, 1997, Bae et al., 2009). IRB is metabolized via oxidative biotransformation and glucuronide conjugation to pharmacologically inactive metabolites (Ruilope, 1997). An in-vitro investigation of influence of hepatic cytochrome P450 isozymes on IRB revealed that the drug is mainly metabolized by CYP2C9, while insignificant metabolism was observed with CYP3A4 isoform (Bourrie et al., 1999). Since, the CYP2C9 isoform of cytochrome is mainly responsible for metabolism of IRB; drugs or herbs that are either substrates or inhibitors may exhibit significant drug-interaction with IRB. One such example is fluconazole, which showed augmentation of C_max_ of IRB by 19% and AUC by 63%, when used concomitantly; however, it did not affect the T_max_ (Kovasc et al., 1999).

Influence of khat on the activity of cytochrome P450 enzyme family have been studied by a number of researchers (Bedada et al., 2015, Bedada et al., 2018, Pantano et al., 2016). In one such study, Lim et al. (2019) reported that the activity of CYP2D9, CYP3A4 and CYP2D6 isoforms was inhibited by khat extract, while cathinone exhibited negligible influence on the tested CYP isoforms. The probability of khat consumption by people taking conventional medications is very high especially in the khat cultivating regions. However, studies investigating khat-drug interactions are limited in the literature. In a previous study, we found that the co-administration of khat and antiplatelet drug clopidogrel resulted in remarkable reduction in the conversion of the drug to its active metabolite in rats, probably by inhibition of CYP 450 isozymes (Alhazmi et al., 2020). The finding of the study indicated urgent need to investigate the interaction of khat with other clinically important drugs that are concordantly being used by regular khat chewers. Among other harmful effects, khat chewing may result in cardiovascular complications and hence, the probability of receiving cardiovascular therapy is greater among the khat chewers. Therefore, evaluation of influence of khat on the pharmacokinetic profile and efficacy of these medications is very important. Owing to inhibitory effects of khat on the activity of various drug-metabolizing isoforms of CYP 450 family, it was expected that the pharmacokinetic profiles of such drugs might get influenced by khat co-administration. Therefore, in the current study, effects of khat leaves chewing on the pharmacokinetic properties of antihypertensive agent, IRB in rats was investigated. The drug was extracted from plasma using simple and fast acetonitrile-based precipitation method and its concentration in rat plasma was monitored using newly developed and validated HPLC method. This study may prove important to draw the attention of clinical practitioners; especially in khat consuming regions towards the probable interaction of khat with other regularly prescribed drugs.

## 2 Materials and methods

### 2.1 Chemicals

IRB (≥98%) and telmisartan (TLN, internal standard, ≥98%) were procured from MedChem Express (USA). Glacial acetic acid and ammonium acetate (LR grade) were purchased from Sigma Aldrich (St. Louis, MO, USA). Acetonitrile and methanol (HPLC-grade) were also purchased from Sigma Aldrich (St. Louis, MO, USA). HPLC- grade water was produced in the laboratory using Milli-Q plus water purification system and used to prepare aqueous solutions (Missouri city, TX, USA).

### 2.2 Plant material

Fresh khat leaves (*Catha edulis*) grown in the southern border region of Jazan Province of Saudi Arabia were selected in the current study. Due to the presence of higher phytochemical contents, newer leaves generally present at the top of the branches were selected. After receiving, fresh leaves were crushed into fine paste.

### 2.3 Animals

Sprague-Dawley (SD) rats weighed between 150 and 300 g were procured from Central Animal House facility of Jazan University, randomized and caged individually in the Pharmacy College facility until study. The room temperature was maintained at 23 ± 2 °C, a relative humidity of 50 ± 10% and light intensity of 150–300 Lux with twelve hour light–dark cycle. Food and water were provided *ad libitum*. The rats were cared according to the standard animal ethics guidelines and used for experiment after taking approval from Standing Committee for Scientific Research Ethics, Jazan University, Saudi Arabia. Animals were fasted overnight before commencement of the experiments.

### 2.4 Preparation of calibration standards and quality control (QC) solutions

The appropriate amounts of IRB and TLN (IS) were weighed and dissolved in methanol to obtain respective stock solutions (1 mg/mL). The stock solutions were further diluted using mobile phase to achieve working standard solution-1 (WS-1, 100 µg/mL) and working standard solution-2 (IRBWS-2, ISWS-2, 20 µg/mL) for both IRB and IS. The stock solutions were stored in refrigerator at 2–8 °C until further used. Measured volumes of IRBWS-2 (25, 50, 100, 200, 300, 400, 500 and 600 μL) were spiked in blank rat plasma to prepare calibration standard solutions having final concentrations in the range of 1–12 µg/mL (7 points). A fixed volume of ISWS-2 (200 μL) was spiked to all calibration and QC solutions to achieve 4 µg/mL concentration and the final volume was adjusted to 1 mL with blank plasma. It was followed by the addition of acetonitrile (1 mL) to precipitate the protein from each solution. The solutions were centrifuged at 5000 rpm for 10 min at 4 °C, supernatants were collected in separate tubes and dried under the stream of N_2_ gas at 40 °C. Each residue was reconstituted in 1 mL of mobile phase and injected into the HPLC system for analysis in triplicate. Three concentrations 1, 4 and 10 µg/mL were used as low quality control (LQC), medium quality control (MQC) and high quality control (HQC) samples, respectively for evaluation of intra- and inter-day precision and accuracy and prepared by following the same procedure as for calibration standards.

### 2.5 Instrumentation and chromatographic conditions

The HPLC system used in this experiment was Waters Breeze series (Waters Corporation, Netherland) consisting of an auto-sampler (2707), a binary pump (1525), and a ultra-violet detector (2489). Analytical separation was performed on basic C-18 column (Raptor C-18, 100 mm × 4.6 mm id.; 5 µm; Restek, USA), which was supported by Raptor C-18 guard column (5 mm × 4.6 mm id.; particle size 5 µm; Restek, USA). The column chamber was maintained at ambient room temperature (25 ± 2 °C). 10 mM ammonium acetate buffer (pH 4.0) and acetonitrile in a ratio of 60:40 *v/v* was used as mobile phase at the flow rate of 1.0 mL/min. The mobile phase was filtered and sonicated for 10 min for complete degassing before use. 10 µL of final sample solutions were injected to achieve separation of IRB and IS using isocratic elution mode. The ultra-violet detector was fixed at 230 nm wavelength and the chromatographic data were acquired and monitored using Waters Breeze (Waters Corporation, Netherland) software.

### 2.6 Pharmacokinetic investigation

Pharmacokinetic study was conducted by randomly dividing the rats into four groups of six animals each. The rats were caged individually. After 12 h of fasting, rats in group I were orally administered with 0.5% sodium carboxy methyl cellulose and served as control group. The rats in group II received IRB (15.5 g/kg body weight) orally; the rats in group III received khat (12.4 g/kg body weight) followed by IRB (15.5 g/kg body weight) orally after 15 min and group IV received only khat (12.4 g/kg body weight) through oral administration. For oral administration to the rats, calculated amount of IRB was suspended in 0.5% sodium carboxy methyl cellulose; while the measured quantity of fresh khat leaves were crushed into fine paste and suspended in water. The doses of IRB and khat leaves were calculated from average daily human intake according to the procedure described in the literature (US, 2005; Shin et al, 2010). Blood samples (approximately 400 µL) from each rat were collected into heparinized tubes at 0, 0.5, 1.0, 2.0, 4.0, 6.0 and 8.0 h time points after IRB administration and an equal volume of saline (50 U/mL) was injected to compensate the blood loss. The collected blood samples were centrifuged at 5000 rpm for 10 min at 4 °C. The supernatant plasma was siphoned off, separated and stored at −80 °C till further use. Prior to injection into the HPLC system for analysis, the plasma stored at −80 °C were thawed and vortexed for 30 s at room temperature. Aliquots (250 µL) from each plasma sample were taken in fresh Eppendorf tubes and fixed volume of IS WS-2 (20 µg/mL) was added to achieve a final concentration of 4 µg/mL and volume was made to 0.5 mL using blank plasma. The mixtures were vortexed again for 30 s followed by addition of 1.5 mL acetonitrile for protein precipitation. The samples were centrifuged at 5000 rpm for 10 min at 4 °C, supernatants were collected in fresh tubes and dried under N_2_ gas stream at 40 °C. The residue thus obtained was reconstituted using 0.125 mL mobile phase, which is half of the actual volume for final reconstitution (0.25 mL). The resulting solutions were filtered through 0.45 µm filter and 10 µL solution was injected into HPLC system. The final concentrations were calculated by multiplying the observed concentration with the dilution factor.

An add-in program for pharmacokinetic data analysis in MS Excel known as PKSolver (Zhang et al., 2010), was used to perform non-compartmental pharmacokinetic data analysis in this study. The linear trapezoidal method was applied to obtain the area under plasma concentration–time graph (AUC), maximum plasma concentration (C_max_) and the time at which C_max_ was recorded (T_max_). The half-life (t_1/2_), volume of distribution (Vd), plasma clearance (CL) and mean-residence time (MRT) were also calculated. Blank plasma was collected from the rats to which no drug was administered and used for preparation and dilution of solutions during method development and validation processes. Data were expressed as Mean ± S.D. for each group and were analyzed using Student’s *t*-test. The *p*-values less than 0.05 were considered statistically significant.

### 2.7 Method validation

The developed HPLC method was evaluated for system suitability, sensitivity, specificity, linearity, accuracy and precision according to ICH and US FDA guidelines for bio-analytical methods validation (​ICH, 2005; ​US FDA, 2018). The system suitability was ascertained by determining USP theoretical plate and tailing factor for analyte peaks, capacity factor, resolution between peaks of IRB and IS and % relative standard deviation (%RSD) of the peak areas of the analyte peaks for six replicate injections of calibration standard (4 µg/mL IRB and IS concentrations). Theoretical plates more than 2000, tailing factor less than 2.0 and the %RSD less than 2.0% were considered as acceptance criteria for system suitability in the current experiment. The specificity was assessed by analyzing blank rat plasma in six replicates with corresponding IRB and IS spiked plasma sample. Method sensitivity was estimated by calculating the limit of detection (LOD) and limit of quantification (LOQ) for IRB. To evaluate the linearity, a series of seven non-zero serially diluted calibration standards with concentrations range from 1 to 12 µg/mL were analyzed in triplicate. The mean peak area of analyte to IS ratios (IRB/IS) were calculated and a calibration curve was constructed by plotting peak area ratio versus corresponding standard concentrations. The least square regression analysis was performed to compute the regression equation, slope and intercept of the calibration curve. A linear fit was proved by finding out the correlation coefficient (R^2^) at 1 – 12 µg/mL concentrations. Intra- and inter-day analyses were performed to establish the precision and accuracy of the present HPLC method. The QC samples at LQC (1 µg/mL), MQC (4 µg/mL) and HQC (10 µg/mL) concentrations were analyzed in six replicates. Intra-day analysis was conducted at three different times on the same day and the QC samples were analyzed at three consecutive days to assess inter-day precision and accuracy. The method precision was expressed by % RSD of the replicate analysis of the QC samples, which should not be more than 15%. Whereas, the accuracy was determined by calculating the % recovery of IRB at each QC concentration, which was determined by comparison of estimated concentration with the nominal concentration. A deviation of less than ± 15% from the nominal concentration at all levels was considered as acceptance criteria. The percent relative standard error (%RE) for IRB at all investigated concentrations of IRB in rat plasma samples was calculated using the following formula:

(1) $$\%RE=\frac{\mathrm{Measured}\;concentration-Nominal\;concentration}{\mathrm{Nominal}\;concentration}\times 100$$

The solution stability of IRB was evaluated at normal laboratory temperature (benchtop condition; 25 ± 2 °C) for 24 h and refrigerator condition (2–8 °C) for 14 days, and three freeze thaw cycles. At the end of each storage condition, solutions (4 μg/mL) were analyzed in triplicate. The percent recovery in each solution was calculated by using peak areas of IRB and IS.

The recovery of IRB and IS was determined by using three QC concentrations separately in five replicates. The recovery values were calculated by comparing the mean peak areas of IRB and IS in QC samples with the mean peak areas of the analytes in samples prepared by spiking into drug-free plasma at same concentrations.

## 3 Results and discussion

### 3.1 HPLC method optimization

**Fig. 2:**
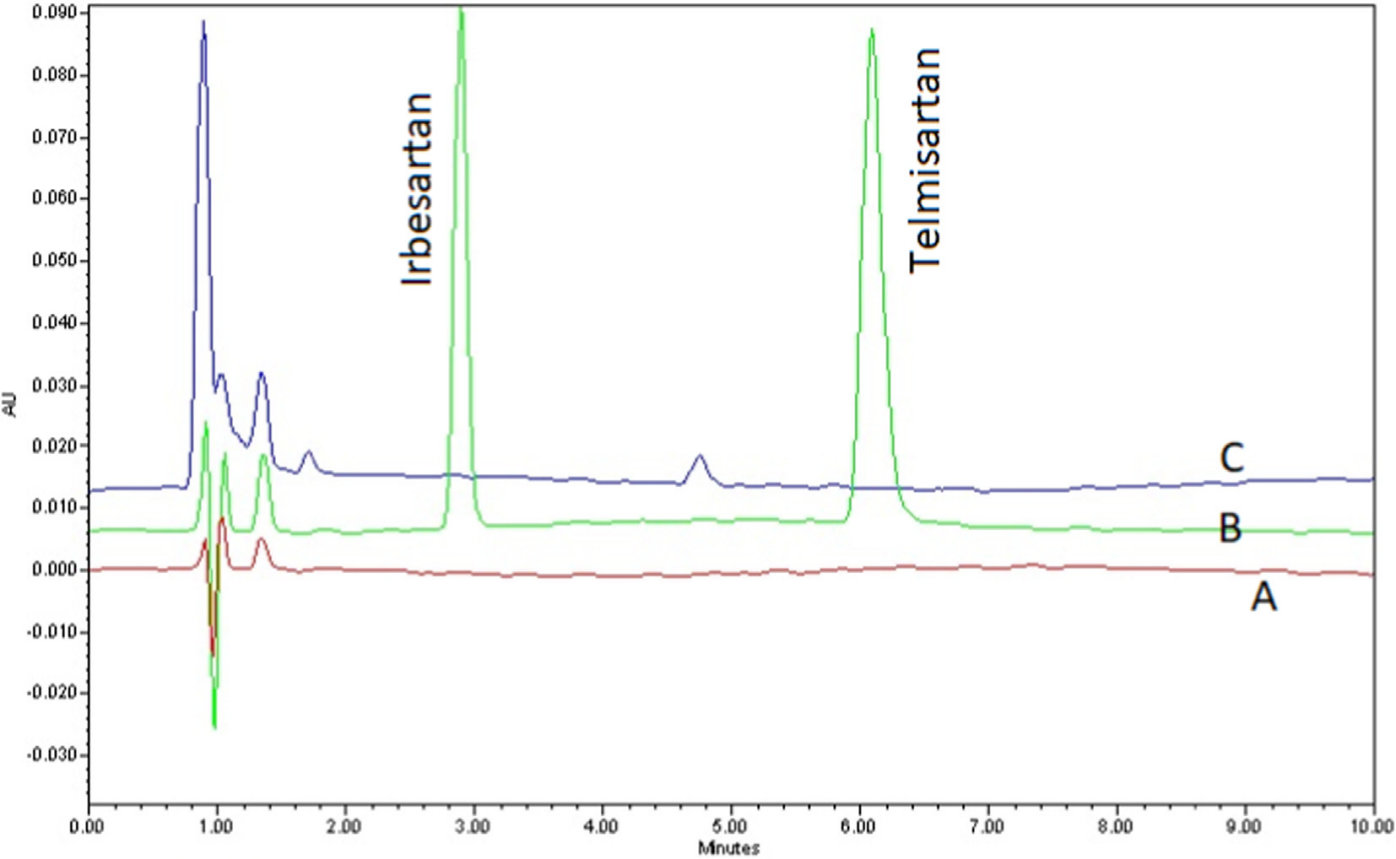
Representative chromatograms of blank (A), IRB (16 µg/mL) and TLN (IS, 4 µg/mL) in rat plasma (B) and blank rat plasma (C). *Chromatographic conditions:* Column-Raptor C-18 (100 mm × 4.6 mm id.; particle size 5 µm); mobile phase-10 mM ammonium acetate buffer pH 4.0 and acetonitrile at a ratio of 60:40 v/v; column temperature-ambient; flow rate-1.0 mL/min; injection volume-10 μL; runtime-10 min and detection wavelength- 230 nm.

The current HPLC method was developed to analyze IRB in rat plasma and measure its concentration with application in pharmacokinetic screening in rats. In order to find out the optimum chromatographic condition for appropriate elution and separation of analyte from IS, various trials were made by applying the chemical and analytical concepts. As both analyte as well as IS were hydrophobic in nature, simple reversed phase C-18 columns were tested to obtain adequate resolution, acceptable run time and symmetric peaks. Enough resolution was obtained on Raptor C-18 column (100 mm × 4.6 mm id.; 5 µm; Restek, USA) supported by Raptor C-18 guard column. After a number of trials using different mobile phase compositions, pH of buffer as aqueous phase and organic modifiers, a mixture of 10 mM ammonium acetate buffer (pH 4.0) and acetonitrile (60:40 *v/v*) was selected as mobile phase for the current analysis. An increase or decrease in the ionic strength of the ammonium acetate buffer or using organic phase other than acetonitrile badly affected the resolution of the analyte. Owing to its simplicity, isocratic elution mode was successfully tried and 1.0 mL/min effluent flow rate was finalized. The optimized chromatographic condition afforded adequate separation and acceptable peak symmetry for IRB and IS. The retention time (RT) for IRB and IS was 2.96 and 6.14 min respectively with a 10 min total run time. Representative chromatograms of IRB and IS is shown in Fig. 2.

### 3.2 Method validation

**Fig. 3:**
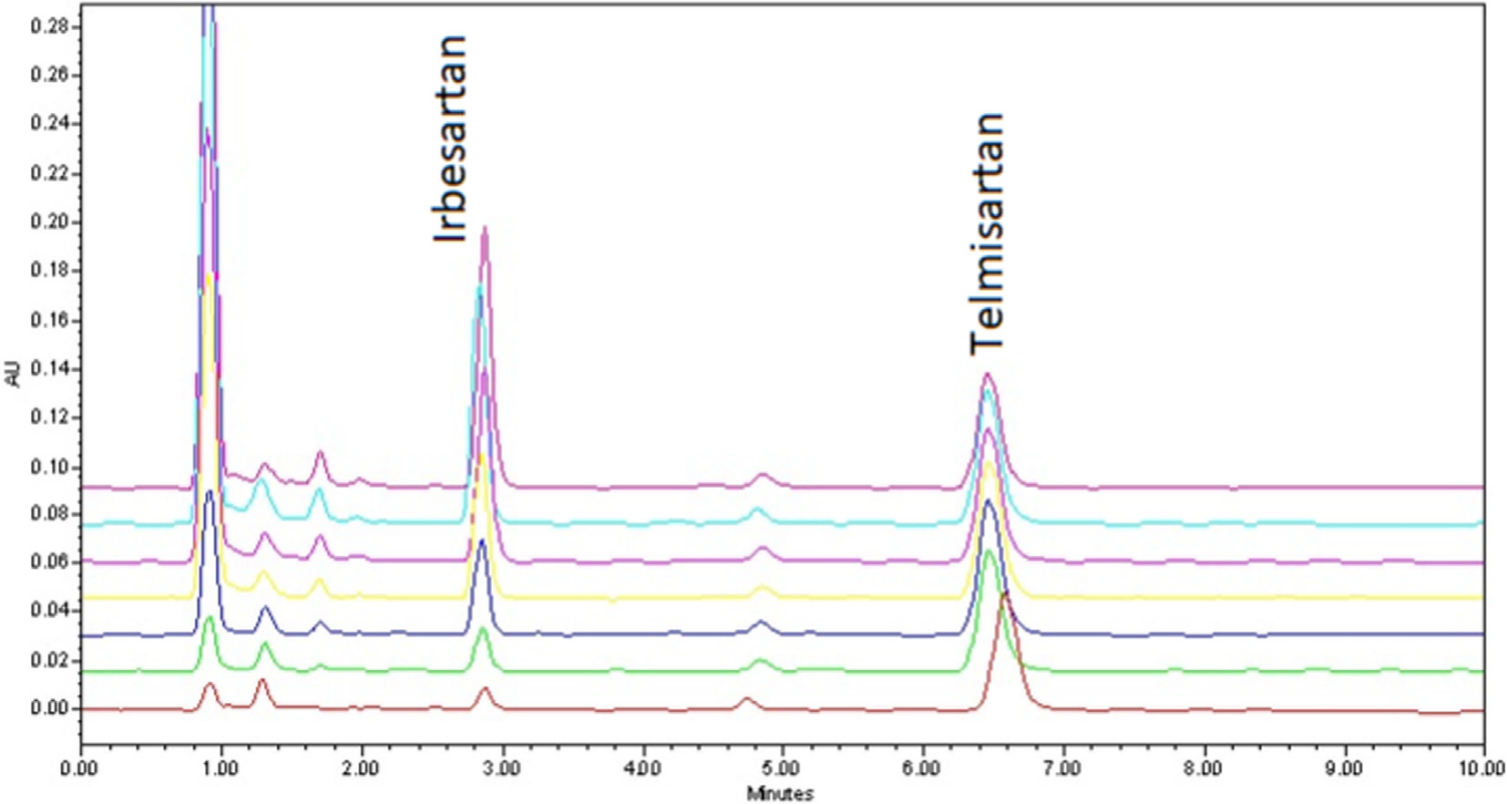
Overlay of chromatograms obtained from analysis of calibration standards with 1–12 μg/mL concentrations (7 points) at 230 nm in rat plasma.

The system suitability parameters calculated in this experiment are summarized in Table 1. The peaks due to IRB and IS were symmetrical (tailing factor less than 2) with acceptable theoretical plate counts. The resolution between the two peaks was satisfactory (> 2) and the % RSD of peak area of six replicate injections were less than 2. The system suitability parameters of the present analysis were within the acceptable limits, indicating that the system was suitable for the analysis of IRB and IS. The specificity of the current HPLC method was established by absence of significant interference from blank and rat plasma matrix in the determination of IRB in plasma sample. In this regard, the chromatograms of spiked rat plasma with IRB and IS, analyte-free rat plasma and blank were evaluated. As evident from Fig. 2, optimum separation and symmetric peak shape of IRB and IS were obtained. Furthermore, no interference from the components of blank and rat plasma at the retention times of analyte and IS was found. The results clearly indicated good specificity of the current method for the bioanalysis of IRB in rat plasma samples. When a series of calibration standards were analyzed under optimized HPLC condition, a linear relationship was recorded over 1 – 12 µg/mL IRB concentration range in the rat plasma. The calibration graph was obtained by plotting peak-area ratio of IRB to IS against drug concentrations with a correlation coefficient (R^2^) > 0.99. The regression equation for IRB was calculated as ‘y = 0.105x – 0.0189′ (n = 7); where, ‘y’ is the peak area ratio of IRB/IS and ‘x’ expresses the drug concentration in rat plasma. An overlay of chromatograms obtained by analyzing calibration standards solution is presented as Fig. 3. The LOD and LOQ values for IRB in plasma samples were found to be 0.103 and 0.345 µg/mL respectively, which indicated acceptable sensitivity of the current bioanalytical method.

**Table 1:** Retention times, tailing factor, resolution, capacity factor and theoretical plate counts values for IRB and TLN (IS) recorded by the developed HPLC method.

Parameters	IRB	TLN
Retention time (min)	2.96	6.14
USP Theoretical plate count	2965	6789
Tailing factor	1.03	1.25
Capacity factor (k)	–	3.74
Resolution	–	3.73
% RSD of peak area (n = 6)	0.61	0.93

The average precision and accuracy results for both intra- and inter-day analyses are summarized in Table 2. Intra-day precision was calculated to be 0.44% − 3.27%; whereas the inter-day precision was recorded in the range of 0.39–1.98% for IRB in plasma samples. The method accuracy was 98.3–102.9% for intra-day analysis and 98.5 – 104.3% for inter-day analysis. The % relative standard error (%RE) was calculated to be −1.67–4.27% for all the investigated concentrations of IRB in plasma samples. The data in Table 2 revealed that both precision and accuracy results were within the acceptable limits for bioanalytical method, indicating reliability and reproducibility of the current method and hence its suitability for the bioanalysis of analyte.

**Table 2:** Intra-day and inter-day-day precision and accuracy results of IRB in rat plasma.

Quality control samples	Intra-day analysis				Inter-day analysis			
	Measured concentration (µg/mL) ± SD	%RSD	% Recovery	%RE	Measured concentration (µg/mL) ± SD	%RSD	% Recovery	%RE
LQC (1.0 µg/mL)	1.03 ± 0.034	3.27	102.9	2.88	1.04 ± 0.021	1.98	104.3	4.27
MQC (4.0 µg/mL)	3.99 ± 0.042	1.06	99.6	−0.36	4.08 ± 0.045	1.11	102.1	2.08
HQC (10.0 µg/mL)	9.83 ± 0.043	0.44	98.3	−1.67	9.85 ± 0.038	0.39	98.5	−1.53

*n = 6

The results of the stability experiment showed that IRB and IS were stable at normal laboratory temperature (benchtop condition; 25 ± 2 °C) for 24 h, refrigerator condition (2–8 °C) for 14 days, and three freeze thaw cycles. The percent recovery of IRB and IS were within 100 ± 2% (Table 3), which indicated that no significant amount of the analytes degraded during the above storage conditions.

**Table 3:** Solution stability data of IRB and telmisartan (IS).

Analytes	Storage conditions (Plasma samples)	Average % recovery*
IRB	Normal lab temperature (25 ± 2 °C) for 24 h	98.69 (±1.023)
	Refrigerator temperature (2–8 °C) for 14 days	99.02 (±0.986)
	Three freeze–thaw cycles	99.12 (±1.023)
IS	Normal lab temperature (25 ± 2 °C) for 24 h	101.16 (±1.261)
	Refrigerator temperature (2–8 °C) for 14 days	99.79 (±0.992)
	Three freeze–thaw cycles	99.82 (±0.925)

*n = 3

The recovery of IRB in plasma ranged from 85.94% to 86.91% and for IS, the recovery was in the ranged from 88.97% to 89.36%. The results were consistent and reproducible at all three concentrations with a RSD of less than 1%. This indicated that the simple protein precipitation method was efficient to extract IRB and IS simultaneously from rat plasma. The recovery results are summarized in Table 4.

**Table 4:** The results of recovery of IRB and IS from rat plasma.

Concentrations of IRB added (µg/mL)	IRB		IS*	
	Recovery (%)**	RSD (%)**	Recovery (%)**	RSD (%)**
1.0	86.23	0.433	89.36	0.231
4.0	86.91	0.619	89.04	0.701
10.0	85.94	0.962	88.97	0.675

*Concentration of IS in all recovery samples was 4 µg/mL.

**n = 5

### 3.3 Determination of IRB in rat plasma

Sample pre-treatment to remove the interfering substances including proteins and other endogenous substances from the sample matrix is very important step in the determination of bioanalytical samples. After generous efforts, protein precipitation using acetonitrile was selected for the current experiment for sample clean-up, which offered a simple method with acceptable recovery of analyte. In the present investigation, 1:1 ratio of acetonitrile to plasma volume was used for optimum results. The applicability of this HPLC method for estimating IRB in plasma sample was proved by acceptable validation results. The developed method showed excellent selectivity and specificity, as no interference from plasma matrix was found at the retention times of IRB and IS. In addition, the method exhibited acceptable precision (%RSD less than 15%) and accuracy (% recovery within 85 – 115%) for IRB in rat plasma samples.

### 3.4 Pharmacokinetic study

**Fig. 4:**
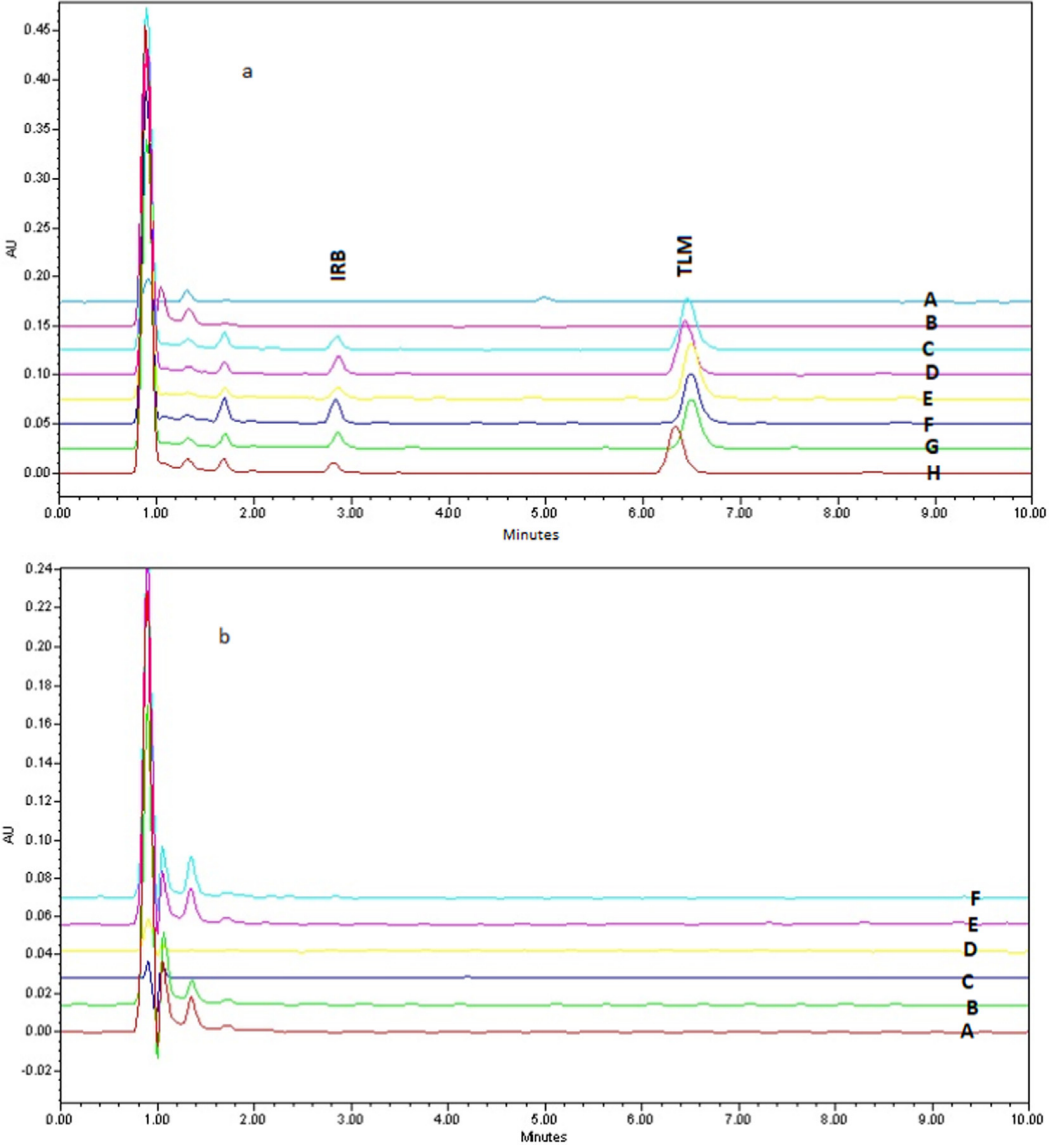
Chromatograms of real samples collected from (a): IRB only administered rats at 0.5, 1, 2, 4, 6 and 8 h (B-H) and blank plasma (A); (b): khat only administered rats at 0.5, 1, 2, 4, 6 and 8 h (A-F), showing no interference at the retention times of IRB and IS.

**Fig. 5:**
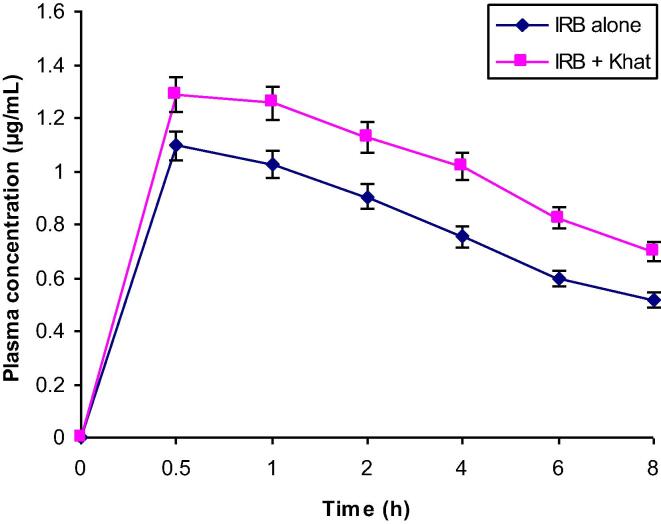
Plasma concentration–time curve obtained after oral administration of single dose of IRB (15.5 mg/kg body weight) in rats with and without pre-administered khat (12.4 mg/kg body weight).

The validated analytical procedures were successfully used for determining IRB concentration in rat plasma samples collected from twelve rats, including six orally administered with IRB alone as single dose of 15.5 mg/kg of body weight (Group II) and the other six administered with IRB (15.5 mg/kg) plus suspension of khat leaves (12.4 mg/kg) (Group III). The specificity of the method in the real sample was established by comparing the chromatograms obtained from group II and group III animals with the chromatograms obtained from rats administered with khat only (Group IV). No interference at the retention times of IRB and IS was observed, indicating acceptable specificity of the method in real sample analysis. Chromatograms of samples with IRB alone and khat alone have been depicted in Fig. 4a and 4b respectively. Various pharmacokinetic parameters were estimated and the effect of khat intake on these parameters was investigated. The mean plasma concentration–time curves for IRB alone and khat + IRB are depicted in Fig. 5 and the important pharmacokinetic parameters calculated by using PKsolver add-in program for IRB with and without khat intake are given in Table 5. Normally, IRB displays unique pharmacokinetic profile amongst angiotensin-II receptor antagonists as it is well-absorbed with 60–80% bioavailability, which is highest among its analogues. In this study, when IRB was administered alone in absence of khat, an AUC_0-t_ of 5.89 ± 0.78 µg h/mL with a maximum concentration (C_max_) of 1.09 ± 0.10 µg/mL was achieved in a short time (T_max_ = 0.5 ± 0.12 h). IRB showed half-life (t_1/2_) of 7.24 h ± 0.43; while, distribution volume (Vd) and plasma clearance (CL) were 14.35 ± 1.21 L and 1.37 ± 0.21 L/h, respectively. On the other hand, when IRB was co-administered with khat leaves, the AUC_0-t_ and C_max_ values increased significantly to 7.66 ± 0.83 µg h/mL and 1.29 ± 0.22 µg/mL, respectively (p<0.05). The highest concentration (C_max_) of drug was observed at 0.5 ± 0.14 h time point (T_max_), which was similar to the khat untreated rats. The plasma half-life (t_1/2_ = 8.44 ± 0.76 h) of IRB + khat further increased in comparison to the IRB only group and comparatively lower value of Vd (11.67 ± 1.32 L) in khat treated group was recorded. Also, the plasma clearance decreased to 0.96 ± 0.28 L/h.

**Table 5:** Pharmacokinetic parameters determined after single oral dose of IRB with and without pre-administration of khat leaves in rat plasma.

Pharmacokinetic parameters	Unit	IRB alone (Group II) (Mean ± SD)	IRB + khat (Group III) (Mean ± SD)
AUC_0-t_	µg h/mL	5.89 ± 0.78	7.66 ± 0.83*
AUC_0-∞_	µg h/mL	11.28 ± 0.49	16.17 ± 1.43
C_max_	µg/mL	1.09 ± 0.10	1.29 ± 0.22*
T_max_	h	0.50 ± 0.12	0.50 ± 0.14
t_1/2_	h	7.24 ± 0.43	8.44 ± 0.76
MRT	h	3.58 ± 0.32	3.69 ± 0.21
Vd	L	14.35 ± 1.21	11.67 ± 1.32
CL	L/h	1.37 ± 0.21	0.96 ± 0.28

Dose of IRB = 15.5 mg/kg; khat = 12.4 mg/kg (fresh leaves)

*p<0.05

As evident from Table 5, the pharmacokinetic parameters, AUC_0-t_ and C_max_ for IRB in IRB + khat co-administered group (III) were greater than those recorded from IRB only treated group (II). The concentration of IRB increased in IRB + khat (III) treated rat plasma in comparison to IRB only administered rats. The AUC_0-t_ enhanced by about 30%, and C_max_ increased by approximately 18%. IRB is frequently used as one of the antihypertensive agents and available in the market mainly in the tablet dosage form. Several researchers reported that the IRB is mainly metabolized by CYP2C9 isoenzyme of cytochrome P450 family (Elgawish et al., 2019, Bae et al., 2009, Borghi et al., 2006); therefore, metabolism of IRB may get affected by the activity of these metabolizing enzymes and CYP450-mediated potential drug-drug interaction may take place. Reports indicating CYP450 inhibitory activity of khat leaves are available and potential khat-drug interaction might occur when the drug is used by a regular khat consumer. In this view, we believed that the co-administration of khat could change the pharmacokinetic profile of IRB by affecting the CYP450 activity. Earlier, we reported a significant decrease in the metabolism of clopidogrel in rats when administered with khat, probably through inhibition of CYP450 enzymes (Alhazmi et al., 2020).

According to a recent study, ethanol extract of khat leaves and small stems inhibited human cytochrome P450 enzymes including CYP2D6, CYP2D9 and CYP34A (Lim et al., 2019). The isoform CYP2D6 was inhibited via competitive and mixed modes; while CPY2D9 isoform got inhibited through both non-competitive and mixed methods, suggesting binding of khat constituents to the sites other than active sites of the enzyme. The extract inhibited CYP3A4 by mixed mode of inhibition only. On the other hand, Cathinone, one of major active constituents of khat did not exhibit any remarkable inhibition of these enzymes (Lim et al., 2019). The above findings indicated the presence of other constituents in the khat extract which are responsible for the enzyme inhibition. Several studies are available that have identified a number of constituents in khat extract including flavonoids, alkaloids, sterols, terpenoids, glycosides, vitamins etc., and many of these constituents were found to exhibit inhibitory effects on various CYP isoforms (Li et al., 2015, Li et al., 1994, Madgula et al., 2009, Pennings et al., 2008, Salminen et al., 2011).

Therefore in the present study, the decrease in the metabolic rate and change in the pharmacokinetic profile of IRB upon co-administration of khat might be attributed to the CYP450 inhibitory activity of khat extract. Results of the current investigation indicated the occurrence of herb-drug interaction; however, clinical investigation is needed to find out the exact influence of khat on IRB metabolism and its consequences on the patient’s health.

## 4 Conclusion

In this study, influence of khat on the pharmacokinetics of IRB, an angiotensin II antagonist was evaluated in rats. Plasma concentration of the drug was determined by newly developed HPLC method, which was validated according to USFDA guidelines with acceptable results. This study showed that the pharmacokinetic parameters of IRB including C_max_ and AUC_0-t_ were significantly increased in khat pre-administered rats as compared to IRB only treated rats, indicating potential drug-herb interaction between IRB and khat. This change in pharmacokinetic profile was probably due to the inhibition of IRB metabolizing enzymes (CYP2D9 isoform of cytochrome P450) by khat. Therefore, physicians and pharmacists should take such drug-herb interaction into consideration in clinical practice and educate the khat users about the possible consequences of khat consumption along with the regular medications.

## Funding

This work was supported by Deanship of Scientific Research, Jazan University, Saudi Arabia under Future Scientist Program [Grant number: FS10-046].

## Ethical approval

The study was approved by the Standing Committee for Scientific Research Ethics (HAPO-10-Z-001), Jazan University [Reference Number: REC42/1/139].

## Authors contributions

The final manuscript was formulated and revised by all the contributors.

## Declaration of Competing Interest

The authors declare that they have no known competing financial interests or personal relationships that could have appeared to influence the work reported in this paper.
